# Implantable Cardioverter–Defibrillator Therapies Following Generator Replacements—Long-Term Remote Monitoring Data

**DOI:** 10.3390/clinpract15090160

**Published:** 2025-08-30

**Authors:** Maciej Dyrbuś, Łukasz Pyka, Anna Kurek, Jacek Niedziela, Elżbieta Adamowicz-Czoch, Katarzyna Sokoła, Joanna Machowicz, Mateusz Ostręga, Damian Pres, Michał Skrzypek, Mariusz Gąsior, Mateusz Tajstra

**Affiliations:** 1 3rd Department of Cardiology, School of Medical Sciences in Zabrze, Medical University of Silesia, 40-055 Katowice, Polandmateusztajstra@wp.pl (M.T.); 2 Department of Biostatistics, Faculty of Public Health in Bytom, Medical University of Silesia, 41-902 Bytom, Poland; mskrzypek@sum.edu.pl

**Keywords:** cardiac resynchronization therapy–defibrillator, generator replacement, implantable cardioverter–defibrillator, remote monitoring

## Abstract

**Background**: The rate of long-term outcomes, including arrhythmic episodes following implantable cardioverter–defibrillator (ICD) device replacements, is often unknown. Thus, the aim of this manuscript was to evaluate the risk of ICD or cardiac resynchronization therapy–defibrillator (CRT-D) therapies in remotely monitored patients following device replacement. **Methods**: Data from 134 patients who underwent ICD/CRT-D replacement or upgrade were analyzed. Kaplan–Meier estimates, as well as Cox proportional hazards regression, were used to present long-term outcomes and predictors of study endpoints, these being all-cause mortality, and appropriate and inappropriate ICD/CRT-D therapies. **Results**: Among the cohort, 51.5% of patients received ICDs and 48.5% received CRT-Ds; the median (quartile 1–quartile 3) LVEF at replacement was 23.0% (18.0–28.0%). In 11 (8.2%) patients, the LVEF at replacement was higher than 35%. During the median (Q1–Q3) follow-up of 3.0 (1.4–5.0) years, 32.1% experienced appropriate and 6.0% experienced inappropriate therapies. The all-cause mortality rate was 38.0%, and appropriate antitachycardia pacing (ATP), a reduced baseline LVEF, and no history of myocardial infarction were independent predictors of death (odds ratios of 1.87 for appropriate ATP, 0.88 per 1% of the LVEF and 0.54 for a history of MI, respectively). The rate of appropriate device therapies was numerically lower in patients whose LVEF improved (19.8% vs. 33.3% and 0% vs. 6.5%, for appropriate and inappropriate therapies). An LVEF of >35% at replacement did not influence the analyzed outcomes. **Conclusions**: In patients who underwent ICD/CRT-D replacement, an improvement in LVEF was not identified as either a predictor of improved survival or of a lower risk of needing device therapies. Further stratification models are needed to evaluate the arrhythmic risk in patients after generator replacements.

## 1. Introduction

Implantable cardioverter–defibrillators (ICDs) are the cornerstone of modern cardiac electrotherapy, improving outcomes in appropriately selected patients with a high risk of sudden cardiac death by treating malignant ventricular arrhythmias, such as ventricular tachycardia or fibrillation. Over the years, significant advances in ICD technology have improved device longevity, programming options, and discrimination algorithms, leading to better patient outcomes and fewer inappropriate therapies [1]. In general, the current ESC guidelines on the prevention of sudden cardiac death recommend the implantation of ICD either in patients who have experienced a sustained ventricular arrhythmia, or (depending on the etiology of heart failure) in patients with a left ventricular ejection fraction below or equal to 35% despite optimal medical treatment [2,3]. As the risk of malignant arrhythmias usually persists in patients in whom an ICD has been implanted as a form of secondary prevention of sudden cardiac death, there is an overall agreement on the need to consecutively replace the generator at the end of the prior device’s life [4]. However, there are no exact guidelines on the optimal strategy for the management of patients in whom an ICD has been implanted as a form of the primary prevention of sudden cardiac death. To date, only a few studies have evaluated the long-term risk of device therapies, and their results are conflicting. Moreover, although the recent BUDAPEST-CRT trial demonstrated that an upgrade to cardiac resynchronization therapy (CRT) allows for a reduction the arrhythmic risk in patients with a high percentage of right ventricular pacing, the real-world data regarding the arrhythmic risk in patients undergoing upgrades to CRT also remain scarce [5]. Remote monitoring allows for continuous connectivity with patients and their implanted devices, providing and allowing the detailed information on device therapies to be stored for further analyses [6,7]. Thus, the aim of the present study was to characterize the rates of ICD use and cardiac resynchronization therapy-defibrillator (CRT-D) therapies in remotely monitored patients who have undergone device replacement or an upgrade from ICD use to CRT-D therapies.

## 2. Materials and Methods

### 2.1. Study Population

As described in detail previously, a total of 1297 patients with an ICD or CRT-D device were subject to remote monitoring (RM) in our facility between 2011 and 2021 [8,9]. RM was allocated to ICD or CRT-D recipients, with eligibility determined by device availability and reimbursement policies. Among these, 295 patients underwent a device replacement or upgrade procedure between October 2012 and December 2021. Of these, 134 patients remained in the RM program after the procedure, while 161 either withdrew or discontinued RM due to a lack of reimbursement for the new RM device, as presented in Supplementary Figure S1. The indications for the replacement were either elective replacement indication (ERI), or hardware issues such as premature battery depletion, while the decision to upgrade the device to CRT-D followed the ESC guidelines on pacing and resynchronization therapy. The choice of the replacement device (ICD or CRT-D) was made at the discretion of the treating and implanting physician and was guided by contemporary ESC recommendations for heart failure management and the prevention of ventricular arrhythmias. Although the study population generally reflects an all-comer group (within the constraints of RM availability and reimbursement), exclusion criteria for participation in the RM program included patients who were unwilling or deemed ineligible to actively undergo RM, as well as those with communication or connectivity issues.

### 2.2. Remote Monitoring

In brief, in the remote monitoring facility of our department, since 2011, dedicated staff members have been continuously monitoring patients with cardiac implantable electronic devices every working day. Data were gathered from RM systems provided by the four major device manufacturers and their respective online platforms. Clinical responses to the transmissions and their outcomes were stored in both paper and electronic formats. As described previously, the remote monitoring registry was created based on the available RM data [9,10,11]. This investigator-initiated, adjudicated, and maintained registry is a single-center, retrospective database encompassing all patients included in the RM program in our department. The registry covers transmission data (including data on both scheduled and alert-triggered transmissions) with a focus on arrhythmic episodes, device interventions (both appropriate and inappropriate), and other hardware- and software-related issues. Patient data are updated annually, and information on arrhythmic episodes and device interventions was drawn from this registry, while the data on the occurrence of hard clinical endpoints, including death, were obtained from the National Health Fund, the sole Polish healthcare provider [11]. For the purpose of the present analysis, the history of the prior devices was obtained (wherever possible) from the patients’ clinical records. Patients were considered for device implantation as a form of secondary prevention of sudden cardiac death when an initial device had already been implanted due to a history of sustained ventricular arrhythmias, when there were appropriate device interventions, or when there had been sustained arrhythmias in the period from the implantation of the prior device to the implantation of the current replacement. The analysis of outcomes was summarized at the one-year mark and across the entire follow-up period, with 30 April 2024 serving as the censoring date. The study was conducted in accordance with the principles of the Declaration of Helsinki, and all patients provided written informed consent to participate in the study.

### 2.3. Statistical Analysis

Descriptive statistics for continuous variables were presented as medians with interquartile ranges (Q1–Q3) for non-normally distributed data or as means with standard deviations (SDs) for normally distributed data, with distribution normality assessed using the Shapiro–Wilk test. Group comparisons were performed using the Mann–Whitney U test for non-normally distributed variables and the *t*-Student test for normally distributed variables. Categorical variables were analyzed using the Pearson’s chi-squared test. Survival analyses were conducted using Kaplan–Meier curves to assess long-term risks of all-cause mortality and device-related therapies. Cox proportional hazards regression models were employed for both univariate and multivariate analyses, with variables selected based on *p* < 0.1 for inclusion and *p* < 0.05 for retention in the final model, to minimize confounders. The variables included in the multivariable analyses are detailed in Supplementary Tables S1–S3, and hazard ratios (HRs) with 95% confidence intervals (CIs) were reported. All analyses were conducted using STATISTICA 10 (StarSoft Inc., Tulsa, OK, USA), with statistical significance set at a two-sided *p*-value of <0.05.

## 3. Results

The clinical characteristics of patients who underwent device replacement and received remote monitoring afterward, in the study period, are summarized in Table 1.

There were 51.5% patients who had an ICD implanted, and in the remaining patients, a CRT-D device was implanted, including both replacements and upgrades. The burden of comorbidities was high, with 43.3% of patients having undergone myocardial infarction or PCI in the past, and 39.6% of patients having diabetes. The median time from the first device’s implantation to the device’s replacement was 4.3 (2.4–5.8) years. The median (quartile 1–quartile 3) values of the LVEF and age at replacement were 23.0% (18.0–28.0%), 64.4 (59.2–69.0) years, respectively. During the median (Q1–Q3) follow-up of 3.0 (1.4–5.0) years, a total of 51 (38.0%) patients died. A total of 43 (32.1%) patients received appropriate therapies during follow-up, and inappropriate defibrillator therapies occurred in 8 (6.0%) of the patients. Among 81.5% of patients with a CRT-D, at least one alert related to low biventricular pacing percentage was reported. There were 114 (85.1%) patients who, at the time of device replacement, had no sustained ventricular arrhythmias in the past, who were considered for replacement as a form of primary prevention of sudden cardiac death. Despite being significantly younger, patients who died had a numerically higher prevalence of diabetes or chronic kidney disease. The LVEF at device implantation and replacement were significantly lower, while left ventricular diameters were significantly larger in subjects who died than those who survived. Among the patients who died, the percentage of subjects who experienced appropriate ATP was significantly higher than that of subjects who survived (41.2% vs. 20.5%, *p* = 0.010). Interestingly, none of the patients who died received any inappropriate therapies, in comparison with eight (9.6%) of the subjects who survived (*p* = 0.024). The comparison of characteristics between patients with regard to LVEF improvement before device replacement is presented in Table 2.

A total of 11 (8.2%) patients had seen an improvement in their LVEF and had the device replaced with an LVEF > 35%. Among them, two had the ICD implanted as a form of secondary prevention of sudden cardiac death. Patients with an improvement in LVEF were more frequently female, had a lower prevalence of comorbidities, including less frequent arterial hypertension, and were older than subjects with a maintained LVEF. Among the patients with an improved LVEF, only two subjects received appropriate ATP, and one subject received appropriate shocks; in none of those patients did any inappropriate therapy occur. The all-cause mortality was numerically higher in patients who underwent any appropriate therapy during follow-up (48.8% vs. 33.0%, *p* = 0.077), as presented in Table 3.

The cohort of patients with appropriate therapies had a significantly more prevalent history of MI (58.1% vs. 36.3%, *p* = 0.017). The LVEF at replacement was numerically lower among patients who received at least one appropriate therapy, with median (Q1–Q3) values of 20% (17.5–26.0%) versus 23% (18.0–30.5%) in subjects with no appropriate therapy during follow-up (*p* = 0.112). A comparison of patients’ characteristics with regard to the occurrence of inappropriate shocks is presented in Supplementary Table S4. In the multivariable analysis, presented in Table 4, the independent predictors of all-cause death after device replacement were a reduction in the baseline LVEF (with each 1% higher LVEF associated with a 12% lower risk of death), no history of myocardial infarction, and the occurrence of any appropriate ATP during follow-up.

**Figure 1:**
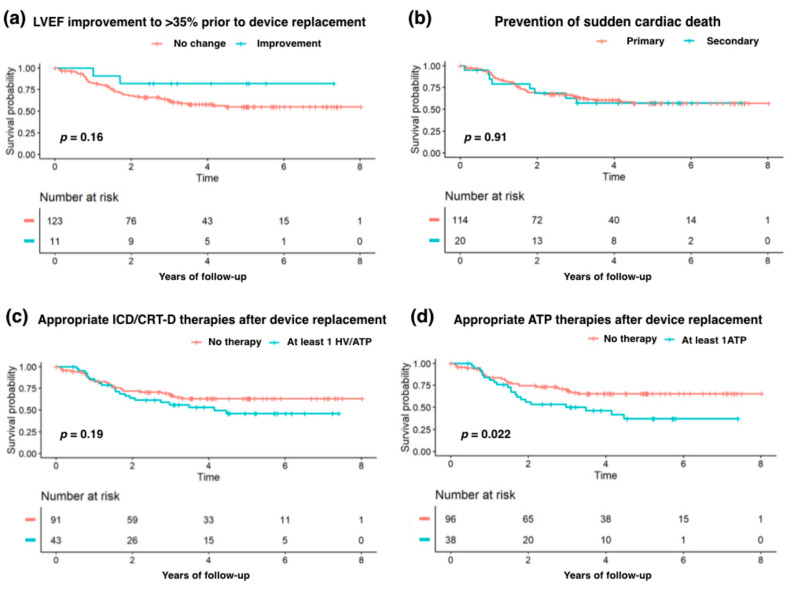
Kaplan–Meier estimates of the long-term risk of all-cause death depending on (**a**) LVEF improvement to >35% prior to device replacement, (**b**) the primary or secondary prevention of sudden cardiac death, (**c**) any appropriate therapies after device replacement, or (**d**) appropriate ATP therapies after replacement. Abbreviations: ATP—antitachycardia pacing; HV—device shock therapy; LVEF—left ventricular ejection fraction. The between-group comparisons were performed with the use of the log-rank tests.

Interestingly, age was an inverted predictor of appropriate and inappropriate ICD/CRT-D therapies, as each year of age, in increasing order, was associated with a 5% lower risk of appropriate therapies and a 13% higher risk of inappropriate therapies after device replacement. The history of myocardial infarction increased the risk of any appropriate ATP by more than twice. The Kaplan–Meier estimates of the cumulative all-cause death risk with regard to LVEF improvement, type of SCD prevention, and the occurrence of either appropriate therapies or appropriate ATP are presented in Figure 1.

## 4. Discussion

The main findings of our study can be summarized as follows: (1) After replacement of their generators, approximately one-third of remotely monitored patients received appropriate therapies due to ventricular arrhythmias. (2) Patients with appropriate therapies had a higher, albeit not statistically significant, all-cause mortality rate, than those who did not receive any appropriate therapies, while appropriate ATP was identified as an independent predictor of all-cause death. (3) Only less than 10% of patients had an LVEF improvement after implantation of their first ICD; however, in those subjects, a numerically lower percentage received an appropriate therapy, while no inappropriate therapies were noted.

The issue of generator replacement has triggered continuous clinical debate, even though the therapy has been a cornerstone of treatment of heart failure, allowing for reductions in the risk of sudden cardiac death, which remains a critical clinical concern [2,3,12,13]. Device replacements have been identified as procedures with an elevated risk of periprocedural complications, including the risk of hematoma and infection [14,15,16]. Although many predictors associated with worse outcomes in patients undergoing device replacement were identified, there is still no consensus on which patients benefit the most from the procedure, and for whom no action should be undertaken [17]. A decision to replace the device is often clinically difficult, especially once the patient potentially loses the indications for an ICD and if they have not suffered from any sustained arrhythmias and thus from any device therapies after the initial implantation. However, an important analysis from the Netherlands indicated that although a majority of ICD patients with devices implanted as a form of primary prevention of sudden cardiac death did not experience malignant arrhythmias, and therapies during the first device’s implantation, 14% of those experienced at least one appropriate therapy after replacement [18]. In the EHRA survey conducted in 2016, the preponderant majority of centers reported either consistent replacement of the ICD, or refraining from replacement only in <10% of cases, pending no ventricular arrhythmias after the prior device’s implantation [4].

There is an increasing number of data pointing out that with the optimal medical therapy offered nowadays, a majority of patients tend to have a significant improvement in their LVEF. In the recent HF-OPT study, 46% of patients with de novo HF had an improvement in their LVEF to >35% at 3 months of optimal treatment, while the rate of patients with an improvement in their LVEF to >35% at 1 year was 77% [19]. On the other hand, a subanalysis of the pivotal SCD-HeFT trial demonstrated that in patients in whom the LVEF improved to >35% after a mean (SD) period of 13 (6) months, the relative reduction in mortality with ICD therapy was comparable to that of patients whose LVEF remained ≤35% [20]. Thus, the question of whether an improvement in LVEF, in the absence of ventricular arrhythmias, is clinically equal to losing an indication for an ICD remains unanswered. Prior trials and registries have shown that the risk of needing ICD therapies declines over time and is most pronounced in the most “acute” period after the onset of heart failure. In our analysis, the risk of appropriate device therapies was high, with almost one-third of patients experiencing either an appropriate shock or an appropriate ATP, even though the included subjects already had their prior devices implanted a median of 5.3 years before.

Although the subgroup of patients whose LVEF increased to >35% was relatively low, our findings indicate that in the long-term follow-up, the risk of therapies in this subgroup was lower, although not negligible. In a recent study by Chang et al., the risk of any appropriate therapy was almost 2.5-fold lower in subjects in whom the LVEF improved to >35%, with 5-year event rates of 12.7% vs. 25.0%, *p* = 0.002, when compared with those in whom the LVEF remained ≤35% [21]. Similarly, in a meta-analysis of six studies, the risk of needing appropriate ICD therapies was almost three-fold higher in subjects whose LVEF remained <35%, or who had a history of appropriate ICD therapies, when compared with patients whose LVEF increased (12.3 vs. 3.4 per 100 person-years) [22]. On the other hand, in the analysis by Looi et al., the risk of appropriate ICD therapies did not differ significantly between patients who still maintained an indication for ICD and those who did not [23]. Although the reported percentages of patients experiencing appropriate therapies are lower than in our population, the differences in clinical characteristics, including a lower LVEF and a high percentage of patients with ischemic cardiomyopathy in our study, could provide at least a partial explanation of this fact. The recent global health events have also highlighted the continued relevance of ICD therapy [24]. During the COVID-19 pandemic, multiple studies reported a decline in the effectiveness and frequency of pre-hospital resuscitation, likely due to delayed emergency responses and reduced bystander CPR [25,26], while the pharmacotherapy strategies during CPR itself are still debatable [27,28].

The findings of our study add to the field of decision making on ICD/CRT-D replacement or upgrade. However, particularly in patients with improved cardiac function, the question of whether all devices should be replaced remains unanswered. While the traditional paradigm has favored routine generator replacement regardless of evolving clinical circumstances, the numerically lower rate of appropriate therapies in patients with an LVEF > 35% raises questions about the necessity of continued defibrillator therapy in this subset of subjects. Although it has been demonstrated that patients who respond to CRT are at lower risk of arrhythmic episodes, this risk does not remain negligible [29]. Thus, it might be that integrated risk stratification scores, based not solely on the LVEF and type of prevention of sudden cardiac death, but also on clinical and imaging data, as well as arrhythmic history, could identify patients at the highest risk of arrhythmic events after generator replacement.

### Limitations

The present study possesses a few limitations that need to be taken into account when analyzing its results. First of all, the analysis is based on retrospective, single-center data, and involves a relatively low number of patients; thus, conclusions on causation should be drawn with caution. However, the study reflects a clinically relevant subgroup of patients undergoing generator replacement with continued remote monitoring. Second, the data regarding the occurrence of arrhythmias and therapies after replacement were obtained from the remote monitoring system, although not all patients had undergone RM prior to ICD/CRT-D replacement; thus, the data on the occurrence of arrhythmic events was solely obtained from patients’ clinical records. Finally, exact data on both prior device detection and therapy zones could not have been obtained in all patients. The issue of device programming is of a major clinical importance, since the more liberal device programming employed in recent years might result in a lower number of registered arrhythmic episodes, thus reducing the number of patients, who in the past years, could have been considered for the secondary prevention of sudden cardiac death due to the occurrence of arrhythmias of a lower frequency than that in currently programmed zones [30].

## 5. Conclusions

Among the patients who had undergone device replacement of implantable cardioverter–defibrillators, or an upgrade or replacement of cardiac resynchronization therapy–defibrillators, the independent predictors of all-cause mortality were baseline left ventricular ejection fraction, a history of myocardial infarction, and the occurrence of any appropriate ATP during post-replacement follow-up. Further stratification models are needed to define the risk of ventricular arrhythmias in patients who have already undergone generator replacements.

## Figures and Tables

**Table 1:** Characteristics of patients who died and survived during the follow-up.

Variable	Overall, N = 134	Survived, N = 83	Died, N = 51	*p*
NYHA class at replacement				0.107
I	19 (17.1%)	13 (17.6%)	6 (16.2%)	
II	46 (41.4%)	35 (47.3%)	11 (29.7%)	
III	42 (37.8%)	25 (33.8%)	17 (45.9%)	
IV	4 (3.6%)	1 (1.4%)	3 (8.1%)	
N/A	23	9	14	
Device after replacement				0.447
ICD	69 (51.5%)	42 (50.6%)	27 (52.9%)	
CRT	49 (36.6%)	33 (39.8%)	16 (31.4%)	
Upgrade to CRT	16 (11.9%)	8 (9.6%)	8 (15.7%)	
Ischemic cardiomyopathy	80 (59.7%)	53 (63.9%)	27 (52.9%)	0.211
Male sex	118 (88.1%)	72 (86.7%)	46 (90.2%)	0.550
Arterial hypertension	66 (49.3%)	48 (57.8%)	18 (35.3%)	0.011
Prior stroke	12 (9.0%)	7 (8.4%)	5 (9.8%)	0.766
Lipid disorders	69 (51.5%)	45 (54.2%)	24 (47.1%)	0.421
History of smoking	48 (35.8%)	32 (38.6%)	16 (31.4%)	0.400
Anemia	33 (24.6%)	20 (24.1%)	13 (25.5%)	0.856
Diabetes	53 (39.6%)	29 (34.9%)	24 (47.1%)	0.164
Chronic kidney disease ≥ 3°	38 (28.4%)	22 (26.5%)	16 (31.4%)	0.544
Atrial fibrillation	53 (39.6%)	35 (42.2%)	18 (35.3%)	0.429
History of PCI	58 (43.3%)	37 (44.6%)	21 (41.2%)	0.700
History of MI	58 (43.3%)	40 (48.2%)	18 (35.3%)	0.143
Secondary prevention of SCD	20 (14.9%)	12 (14.5%)	8 (15.7%)	0.846
Appropriate shocks	33 (24.6%)	17 (20.5%)	16 (31.4%)	0.155
Inappropriate shocks	5 (3.7%)	5 (6.0%)	0 (0.0%)	0.156
Appropriate ATP	38 (28.4%)	17 (20.5%)	21 (41.2%)	0.010
Inappropriate ATP	7 (5.2%)	7 (8.4%)	0 (0.0%)	0.044
Appropriate therapies	43 (32.1%)	22 (26.5%)	21 (41.2%)	0.077
Inappropriate therapies	8 (6.0%)	8 (9.6%)	0 (0.0%)	0.024
Low %BiV (during follow-up)	53/65 (81.5%)	31/41 (75.6%)	22/24 (91.7%)	0.107
LVEF at replacement	23.00 (18.00, 28.00)	25.00 (19.00–32.00)	20.00 (17.00–25.00)	0.003
Age	64.39 (59.22, 68.99)	65.63 (8.39)	61.68 (7.84)	0.008
LVEDD at implantation	68.80 (9.92)	66.00 (60.00–71.00)	72.00 (65.00–79.00)	0.001
LVESD at implantation	56.76 (11.78)	53.66 (11.86)	61.43 (10.08)	<0.001
LVEF at implantation	24.00 (20.00, 28.00)	25.00 (22.00–30.00)	20.00 (17.00–25.00)	<0.001

Abbreviations: ATP—antitachycardia pacing; BiV—biventricular; CRT—cardiac resynchronization therapy; ICD—implantable cardioverter defibrillator; LVEDD—left ventricular end-diastolic diameter; LVEF—left ventricular ejection fraction; LVESD—left ventricular end-systolic diameter; MI—myocardial infarction; NYHA—New York Heart Association; PCI—percutaneous coronary intervention; SCD—sudden cardiac death.

**Table 2:** Characteristics of patients with regard to improvements in LVEF when compared with those upon implantation of the prior device.

Variable	Overall, N = 134	No Improvement of LVEF to >35%, N = 123	Improvement of LVEF to >35%, N = 11	*p*-Value
All-cause death	51 (38.1%)	49 (39.8%)	2 (18.2%)	0.205
NYHA class at replacement				0.483
I	19 (17.1%)	16 (15.7%)	3 (33.3%)	
II	46 (41.4%)	42 (41.2%)	4 (44.4%)	
III	42 (37.8%)	40 (39.2%)	2 (22.2%)	
IV	4 (3.6%)	4 (3.9%)	0 (0.0%)	
No data	23	21	2	
Device after replacement				0.155
ICD	69 (51.5%)	65 (52.8%)	4 (36.4%)	
CRT	49 (36.6%)	42 (34.1%)	7 (63.6%)	
Upgrade	16 (11.9%)	16 (13.0%)	0 (0.0%)	
Ischemic cardiomyopathy	80 (59.7%)	74 (60.2%)	6 (54.5%)	0.756
Male sex	118 (88.1%)	111 (90.2%)	7 (63.6%)	0.027
Arterial hypertension	66 (49.3%)	64 (52.0%)	2 (18.2%)	0.031
Prior stroke	12 (9.0%)	12 (9.8%)	0 (0.0%)	0.598
Lipid disorders	69 (51.5%)	63 (51.2%)	6 (54.5%)	0.833
History of smoking	48 (35.8%)	44 (35.8%)	4 (36.4%)	>0.999
anemia	33 (24.6%)	30 (24.4%)	3 (27.3%)	>0.999
Diabetes	53 (39.6%)	50 (40.7%)	3 (27.3%)	0.526
Chronic kidney disease ≥ 3°	38 (28.4%)	35 (28.5%)	3 (27.3%)	>0.999
Atrial fibrillation	53 (39.6%)	49 (39.8%)	4 (36.4%)	>0.999
History of PCI	58 (43.3%)	54 (43.9%)	4 (36.4%)	0.756
History of MI	58 (43.3%)	55 (44.7%)	3 (27.3%)	0.349
Secondary prevention of SCD	20 (14.9%)	18 (14.6%)	2 (18.2%)	0.669
Appropriate shocks	33 (24.6%)	32 (26.0%)	1 (9.1%)	0.292
Inappropriate shocks	5 (3.7%)	5 (4.1%)	0 (0.0%)	>0.999
Appropriate ATP	38 (28.4%)	36 (29.3%)	2 (18.2%)	0.728
Inappropriate ATP	7 (5.2%)	7 (5.7%)	0 (0.0%)	>0.999
Appropriate therapies	43 (32.1%)	41 (33.3%)	2 (19.8%)	0.105
Inappropriate therapies	8 (6.0%)	8 (6.5%)	0 (0.0%)	>0.999
Low %BiV alert (during follow-up)	53/65 (81.5%)	49/58 (81.5%)	4/7 (57.1%)	0.118
LVEF at replacement	23.00 (18.00, 28.00)	21.00 (18.00, 27.00)	45.00 (41.50, 45.00)	<0.001
Age at replacement	64.39 (59.22, 68.99)	63.44 (58.83, 68.40)	67.81 (65.87, 79.31)	0.011
LVEDD at implantation	68.80 (9.92)	69.55 (9.71)	60.64 (8.82)	0.008
LVESD at implantation	56.76 (11.78)	57.68 (11.55)	47.00 (9.98)	0.005
LVEF at implantation	24.00 (20.00, 28.00)	23.00 (19.00, 28.00)	28.00 (25.00, 31.00)	0.032

Abbreviations: ATP—antitachycardia pacing; BiV—biventricular; CRT—cardiac resynchronization therapy; ICD—implantable cardioverter defibrillator; LVEDD—left ventricular end-diastolic diameter; LVEF—left ventricular ejection fraction; LVESD—left ventricular end-systolic diameter; MI—myocardial infarction; NYHA—New York Heart Association; PCI—percutaneous coronary intervention; SCD—sudden cardiac death.

**Table 3:** Characteristics of patients with regard to the occurrence of any appropriate therapies.

Variable	Overall, N = 134	No Appropriate Therapy During Follow-Up, N = 91	At Least One Appropriate Therapy, N = 43	*p*-Value
All-cause death	51 (38.1%)	30 (33.0%)	21 (48.8%)	0.077
NYHA class at replacement				>0.999
I	19 (17.1%)	13 (16.9%)	6 (17.6%)	
II	46 (41.4%)	32 (41.6%)	14 (41.2%)	
III	42 (37.8%)	29 (37.7%)	13 (38.2%)	
IV	4 (3.6%)	3 (3.9%)	1 (2.9%)	
N/A	23	14	9	
Device after replacement				0.160
ICD	69 (51.5%)	42 (46.2%)	27 (62.8%)	
CRT	49 (36.6%)	38 (41.8%)	11 (25.6%)	
Upgrade	16 (11.9%)	11 (12.1%)	5 (11.6%)	
Ischemic cardiomyopathy	80 (59.7%)	50 (54.9%)	30 (69.8%)	0.102
Male sex	118 (88.1%)	78 (85.7%)	40 (93.0%)	0.223
Arterial hypertension	66 (49.3%)	46 (50.5%)	20 (46.5%)	0.663
Prior stroke	12 (9.0%)	9 (9.9%)	3 (7.0%)	0.751
Lipid disorders	69 (51.5%)	47 (51.6%)	22 (51.2%)	0.958
History of smoking	48 (35.8%)	33 (36.3%)	15 (34.9%)	0.876
Anemia	33 (24.6%)	23 (25.3%)	10 (23.3%)	0.800
Diabetes	53 (39.6%)	40 (44.0%)	13 (30.2%)	0.129
Chronic kidney disease ≥ 3°	38 (28.4%)	28 (30.8%)	10 (23.3%)	0.368
Atrial fibrillation	53 (39.6%)	36 (39.6%)	17 (39.5%)	0.998
History of PCI	58 (43.3%)	36 (39.6%)	22 (51.2%)	0.206
History of MI	58 (43.3%)	33 (36.3%)	25 (58.1%)	0.017
Secondary prevention of SCD	20 (14.9%)	14 (15.4%)	6 (14.0%)	0.828
LVEF change after first device implantation				0.105
LVEF remaining ≤35%	123 (91.8%)	81 (89.0%)	42 (97.7%)	
Improvement to >35%	11 (8.2%)	10 (11.0%)	1 (2.3%)	
Inappropriate shocks	5 (3.7%)	4 (4.4%)	1 (2.3%)	>0.999
Inappropriate ATP	7 (5.2%)	5 (5.5%)	2 (4.7%)	>0.999
Inappropriate therapies	8 (6.0%)	6 (6.6%)	2 (4.7%)	>0.999
Low %BiV alert (during follow-up)	53 (39.6%)	31 (34.1%)	22 (51.2%)	0.059
LVEF at replacement	23.00 (18.00, 28.00)	23.00 (18.00, 30.50)	20.00 (17.50, 26.00)	0.112
Age	64.13 (8.38)	64.94 (8.54)	62.40 (7.85)	0.092
LVEDD at implantation	68.80 (9.92)	67.80 (9.77)	70.88 (10.01)	0.098
LVESD at implantation	56.76 (11.78)	55.34 (11.78)	59.67 (11.38)	0.049
LVEF at implantation	24.00 (20.00, 28.00)	25.00 (20.00, 30.00)	22.00 (18.00, 28.00)	0.047

Abbreviations: ATP—antitachycardia pacing; BiV—biventricular; CRT—cardiac resynchronization therapy; ICD—implantable cardioverter defibrillator; LVEDD—left ventricular end-diastolic diameter; LVEF—left ventricular ejection fraction; LVESD—left ventricular end-systolic diameter; MI—myocardial infarction; NYHA—New York Heart Association; PCI—percutaneous coronary intervention; SCD—sudden cardiac death.

**Table 4:** Predictors of death, appropriate, and inappropriate ICD/CRT-D therapies after device replacement based on multivariable Cox regression.

	HR	95% CI	*p*-Value
Independent Predictors of Death			
Baseline LVEF, per 1% increase	0.88	0.84, 0.94	<0.001
History of myocardial infarction	0.54	0.30, 0.98	0.042
Appropriate ATP during follow-up	1.87	1.05, 3.30	0.032
Independent predictors of appropriate therapies after device replacement			
Age, per 1 year	0.95	0.92, 0.98	0.005
History of myocardial infarction	2.22	1.19, 4.15	0.012
Independent predictors of inappropriate therapies after device replacement			
Age, per 1 year	1.13	1.02, 1.24	0.018

Abbreviations: ATP—antitachycardia pacing; CI—confidence interval; HR—hazard ratio; LVEF—left ventricular ejection fraction.

## Data Availability

The source data that support the findings of this study are available from the corresponding author, [M.D.], upon request.

## Supplementary Materials

The following supporting information can be downloaded: https://www.mdpi.com/article/10.3390/clinpract15090160/s1. Figure S1: Study flowchart. Table S1: Results of univariable regression regarding the risk of death after device replacement. Table S2: Results of univariable regression regarding the risk of appropriate ICD/CRT-D therapies after device replacement. Table S3: Results of univariable regression regarding the risk of inappropriate ICD/CRT-D therapies after device replacement. Table S4: Characteristics of patients with regard to the occurrence of any inappropriate therapies.

## Abbreviations

The following abbreviations are used in this manuscript:

CRT-D	Cardiac resynchronization therapy–defibrillator
HF	Heart failure
ICD	Implantable cardioverter–defibrillator
LVEF	Left ventricular ejection fraction
ATP	Antitachycardia pacing
