# Supplementary material for: Implantable Cardioverter–Defibrillator Therapies Following Generator Replacements—Long-Term Remote Monitoring Data
Source: Clin Pract. 2025 Aug 30;15(9):160. doi: 10.3390/clinpract15090160 (PMC12468052; doi:10.3390/clinpract15090160)

Supplementary Table S1: Results of univariable regression regarding the risk of death after device replacement

**Predictor**	**HR**	**95% CI**	**P value**
LVEF at replacement	0.943	0.904; 0.982	0.0049
Age	0.960	0.931; 0.99	0.0087
LVEDD at implantation	1.035	1.009; 1.062	0.0077
LVESD at implantation	1.041	1.017; 1.065	0.0007
LVEF at implantation	0.888	0.841; 0.937	<.0001
NYHA class at replacement II vs I	0.757	0.28; 2.047	0.5827
NYHA class at replacement III vs I	1.368	0.539; 3.475	0.5095
NYHA class at replacement IV vs I	3.091	0.772; 12.377	0.1108
CRT-D vs ICD post replacement	1.201	0.647; 2.23	0.5612
Ischemic cardiomyopathy	0.696	0.401; 1.207	0.1968
Male sex	1.140	0.453; 2.87	0.7808
Hypertension	0.485	0.273; 0.863	0.0138
History of stroke	1.255	0.498; 3.158	0.6303
Hyperlipidemia	0.778	0.448; 1.348	0.3703
Smoking history	0.721	0.399; 1.304	0.2796
Anaemia	1.027	0.547; 1.929	0.9332
Diabetes mellitus	1.557	0.898; 2.699	0.1150
Chronic kidney disease ≥3º	1.164	0.644; 2.103	0.6146
Atrial fibrillation	0.844	0.475; 1.499	0.5624
History of PCI	0.804	0.46; 1.405	0.4437
History of MI	0.651	0.367; 1.157	0.1433
Secondary prevention of SCD	1.044	0.491; 2.221	0.9109
Increase of LVEF to >35%	0.378	0.092; 1.554	0.1775
Appropriate shocks after replacement	1.398	0.774; 2.526	0.2670
Appropriate ATP after replacement	1.903	1.088; 3.327	0.0241
Appropriate therapies after replacement	1.448	0.829; 2.53	0.1930

Abbreviations: ATP – Antitachycardia Pacing; BiV – Biventricular; CRT – Cardiac Resynchronization Therapy; ICD – Implantable Cardioverter Defibrillator; LVEDD – Left Ventricular End-Diastolic Diameter; LVEF – Left Ventricular Ejection Fraction; LVESD – Left Ventricular End-Systolic Diameter; MI – Myocardial Infarction; NYHA – New York Heart Association; PCI – Percutaneous Coronary Intervention; SCD – Sudden Cardiac Death.

Supplementary Table S2: Results of univariable regression regarding the risk of appropriate ICD/CRT-D therapies after device replacement

Predictor	HR	95% CI	P value
LVEF at replacement	0.952	0.913; 0.992	0.0206
Age	0.959	0.927; 0.993	0.0182
LVEDD at implantation	1.035	1.005; 1.065	0.0202
LVESD at implantation	1.034	1.007; 1.062	0.0145
LVEF at implantation	0.921	0.872; 0.973	0.0031
NYHA class at replacement II vs I	1.192	0.457; 3.108	0.7196
NYHA class at replacement III vs I	1.221	0.463; 3.221	0.6865
NYHA class at replacement IV vs I	0.901	0.108; 7.534	0.9237
CRT-D vs ICD post replacement	1.862	0.921; 3.764	0.0834
Ischemic cardiomyopathy	1.609	0.838; 3.086	0.1528
Male sex	1.762	0.544; 5.71	0.3449
Hypertension	0.832	0.454; 1.524	0.5514
History of stroke	0.849	0.262; 2.746	0.7842
Hyperlipidemia	0.993	0.546; 1.808	0.9825
Smoking history	0.839	0.448; 1.572	0.5833
Anaemia	0.953	0.469; 1.937	0.8952
Diabetes mellitus	0.755	0.393; 1.452	0.3996
Chronic kidney disease ≥3°	0.696	0.343; 1.413	0.3161
Atrial fibrillation	0.906	0.491; 1.673	0.7534
History of PCI	1.438	0.788; 2.624	0.2366
History of MI	1.857	1.012; 3.409	0.0457
Secondary prevention of SCD	0.848	0.358; 2.011	0.7085
Increase of LVEF to >35%	0.212	0.029; 1.543	0.1256

Supplementary Table S3: Results of univariable regression regarding the risk of inappropriate ICD/CRT-D therapies after device replacement

**Predictor**	**HR**	**95% CI**	**P value**
LVEF at replacement	0.999	0.92; 1.085	0.9812
Age	1.097	1.009; 1.193	0.0308
LVEDD at implantation	0.963	0.893; 1.038	0.3211
LVESD at implantation	0.963	0.906; 1.023	0.2254
LVEF at implantation	1.094	0.961; 1.244	0.1733
NYHA class at replacement II vs I	1.232	0.128; 11.853	0.8567
NYHA class at replacement III vs I	1.549	0.16; 14.987	0.7054
CRT-D vs ICD post replacement	1.170	0.28; 4.898	0.8297
Ischemic cardiomyopathy	0.604	0.15; 2.422	0.4765
Male sex	0.354	0.071; 1.763	0.2051
Hypertension	1.478	0.352; 6.21	0.5937
History of stroke	1.433	0.176; 11.647	0.7366
Hyperlipidemia	0.858	0.214; 3.435	0.8287
Smoking history	0.960	0.229; 4.021	0.9555
Anaemia	0.962	0.194; 4.779	0.9624
Diabetes mellitus	0.549	0.111; 2.721	0.4626
Chronic kidney disease ≥3°	2.748	0.686; 11.015	0.1535
Atrial fibrillation	2.588	0.618; 10.838	0.1931
History of PCI	0.675	0.161; 2.835	0.5915
History of MI	0.167	0.02; 1.357	0.0940
Secondary prevention of SCD	1.761	0.355; 8.738	0.4884

Supplementary Table S4: **Characteristics of patients with regard to the occurrence of any inappropriate therapies**

**Variable**	**Overall, N = 134**	**No inappropriate therapy during follow-up, N = 126**	**At least one inappropriate therapy at follow-up, N = 8**	**p-value**
All-cause death	51 (38.1%)	51 (40.5%)	0 (0.0%)	**0.024**
NYHA class at replacement				>0.999
1	19 (17.1%)	18 (17.3%)	1 (14.3%)	
2	46 (41.4%)	43 (41.3%)	3 (42.9%)	
3	42 (37.8%)	39 (37.5%)	3 (42.9%)	
4	4 (3.6%)	4 (3.8%)	0 (0.0%)	
N/A	23	22	1	
Device after replacement				0.776
ICD	69 (51.5%)	64 (50.8%)	5 (62.5%)	
CRT	49 (36.6%)	46 (36.5%)	3 (37.5%)	
Upgrade	16 (11.9%)	16 (12.7%)	0 (0.0%)	
Ischemic cardiomyopathy	80 (59.7%)	76 (60.3%)	4 (50.0%)	0.714
Male sex	118 (88.1%)	112 (88.9%)	6 (75.0%)	0.244
Arterial hypertension	66 (49.3%)	61 (48.4%)	5 (62.5%)	0.489
Prior stroke	12 (9.0%)	11 (8.7%)	1 (12.5%)	0.538
Lipid disorders	69 (51.5%)	65 (51.6%)	4 (50.0%)	>0.999
History of smoking	48 (35.8%)	45 (35.7%)	3 (37.5%)	>0.999
Anaemia	33 (24.6%)	31 (24.6%)	2 (25.0%)	>0.999
Diabetes	53 (39.6%)	51 (40.5%)	2 (25.0%)	0.478
Chronic kidney disease ≥3°	38 (28.4%)	34 (27.0%)	4 (50.0%)	0.222
Atrial fibrillation	53 (39.6%)	48 (38.1%)	5 (62.5%)	0.264
History of PCI	58 (43.3%)	55 (43.7%)	3 (37.5%)	>0.999
History of MI	58 (43.3%)	57 (45.2%)	1 (12.5%)	0.137
Secondary prevention of SCD	20 (14.9%)	18 (14.3%)	2 (25.0%)	0.341
LVEF change after first device implantation				>0.999
LVEF remaining ≤35%	123 (91.8%)	115 (91.3%)	8 (100.0%)	
Improvement to >35%	11 (8.2%)	11 (8.7%)	0 (0.0%)	
Appropriate shocks	33 (24.6%)	32 (25.4%)	1 (12.5%)	0.679
Appropriate ATP	38 (28.4%)	36 (28.6%)	2 (25.0%)	>0.999
Appropriate therapies	43 (32.1%)	41 (32.5%)	2 (25.0%)	>0.999
Low %BiV alert (during follow-up)	53 (39.6%)	49 (38.9%)	4 (50.0%)	0.712
LVEF at replacement	23.00 (18.00, 28.00)	22.00 (18.00, 28.00)	24.50 (22.00, 28.50)	0.592
Age	64.13 (8.38)	63.70 (8.31)	70.87 (6.70)	**0.019**
LVEDD at implantation	68.80 (9.92)	69.07 (10.08)	64.63 (5.78)	0.075
LVESD at implantation	56.76 (11.78)	57.11 (11.81)	50.71 (10.13)	0.152
LVEF at implantation	24.00 (20.00, 28.00)	23.00 (19.25, 28.00)	27.00 (24.75, 30.75)	0.095

Supplementary Figure S1: Study flowchart

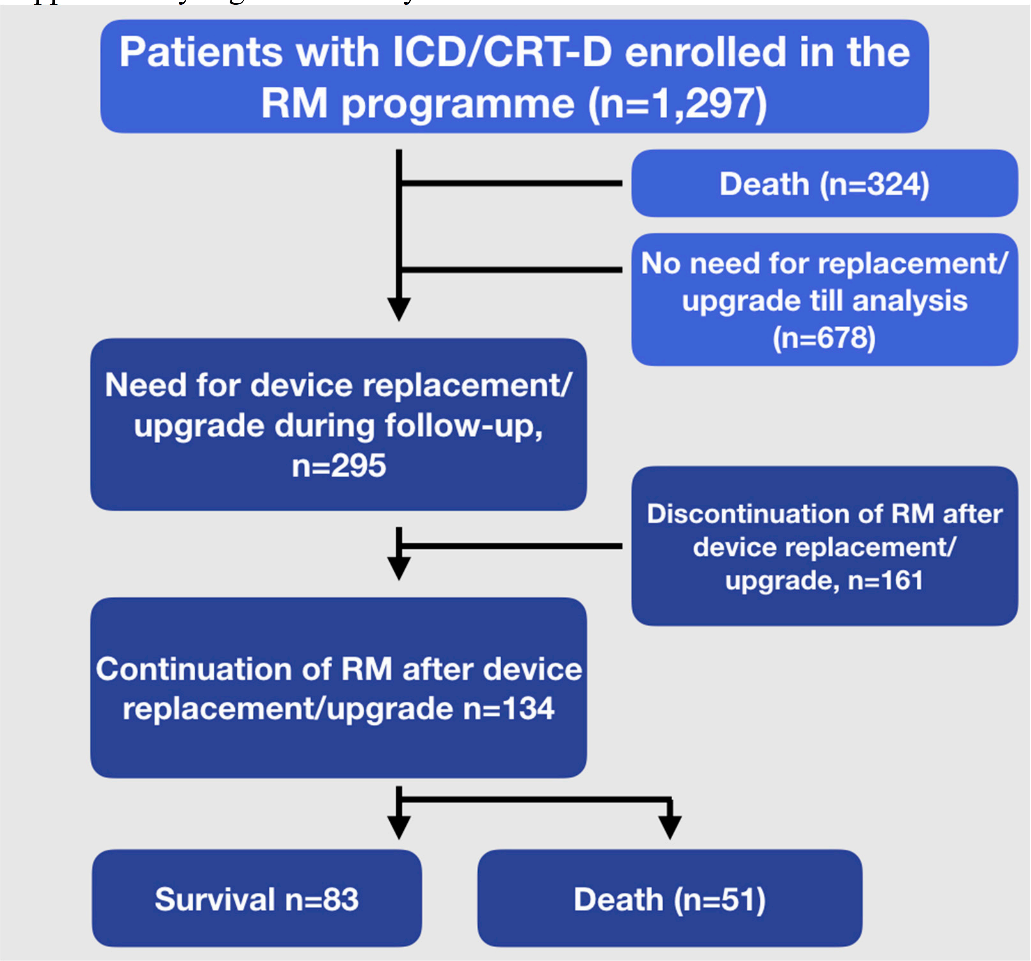

Supplement: Supplementary file 1 [file clinpract-15-00160-s001.zip › clinpract-3438088-supplementary.pdf]
